# The Daily Rhythmic Changes of Undergraduate Students’ Emotions: An Analysis Based on Tencent Tweets

**DOI:** 10.3389/fpsyg.2022.785639

**Published:** 2022-03-11

**Authors:** Run-Xiang Liu, Huan Liu

**Affiliations:** ^1^Department of Psychology, School of Public Policy and Administration, Nanchang University, Nanchang, China; ^2^Mental Health Education Centre, Nanchang University, Nanchang, China

**Keywords:** emotion, daily rhythm, text analysis, Tencent tweets (TTs), undergraduate student

## Abstract

Emotional stability is of great importance for undergraduates and has significant predictive power for mental health. Emotions are associated with individuals’ daily lives and routines. Undergraduates commonly post their opinions and feelings on social networks, providing a huge amount of data for studying their emotional states and rhythms. Based on the construction of the emotion dictionary of undergraduates’ Tencent tweets (TTs)—a social network for users to share their life situations and express emotions and feelings to friends—we used big data text analysis technology to analyze the emotion words in 45,996 Tencent tweets published by 894 undergraduates. Then, we used hierarchical linear modeling to further analyze the daily rhythms of undergraduate students’ emotions and how demographic variables are associated with the daily rhythmic changes. The results were as follows: (1) Undergraduates tweeted about more positive emotions than negative emotions (love was most common and fear was the least common); (2) The emotions in undergraduates’ tweets changed considerably from 1 a.m. to 6 a.m., but were fairly stable during the day; (3) There was a rising trend in the frequency of using emotion words in Tencent tweets during the day as each hour progressed, and there was a higher increase in positive emotion than negative emotion; and (4) The word frequencies and daily rhythms of emotions varied depending on demographic variables. Gender was correlated with the frequencies of gratitude and the daily rhythms of anger. As the grade increased, the frequency of emotion words in most subcategories in TTs decreased and the fluctuation in daily rhythms became smaller. There was no significant difference in the frequency and daily rhythm of emotion words used in TTs based on having had a left-behind experience. The results of the present study provided emotion expression in social networks in Chinese collectivist culture. This study added new evidence to support the notion that positive and negative emotions are independent dimensions.

## Introduction

Undergraduate students are in the critical period of developing self-identity; they face many challenges in academics, socializing, career planning, and intimate relationships, and their emotions are changeable and unstable. Recent research has indicated that emotional stability has significant predictive power over mental health. Emotional instability occurs in a wide range of mental disorders (Patel et al., 2015). In contrast, individuals with stable emotions reported a high degree of experienced happiness and life satisfaction (Hills and Argyle, 2001; Bajaj et al., 2019). Therefore, emotional stability is of great importance when considering improving mental health.

Individuals’ emotions change with their daily lives and interactions, and this has a biological basis. The suprachiasmatic nucleus regulates the circadian rhythm. The suprachiasmatic nucleus maintains the internal rhythm and keeps the same pace as the outer environment; this is done by receiving photic and non-photic cues and integrating them with the internal status. When suprachiasmatic nucleus rhythms are perturbed by genetic or environmental disturbances, brain regions related to mood regulation may be disrupted, leading to emotional disorders (Vadnie and McClung, 2017). In addition to the suprachiasmatic nucleus, individuals’ emotions may be disturbed by the circadian rhythm through the immune system, monoamine transportation, metabolic meditation, neuron regeneration, and other systems (McClung, 2013).

The daily rhythms of emotion are also associated with individuals’ daily routines and habits. Golder and Macy (2011) found that positive emotions rose in the mornings and decreased after midnight, while negative emotions remained high throughout the night. Moreover, for “night owls,” who slept during the day and were awake at night, their daily rhythms of emotions were different from those people with ordinary routines. Wang et al.’s (2016) study showed similar results. Bellivier et al. (2015) also found that almost all individuals with emotional disorders have an obvious daily rhythmic disturbance. Meanwhile, they found that disturbed circadian rhythms are closely associated with emotional fluctuations and disorders. Emotional fluctuations and disorders are more likely to emerge or further deepen in those who undertake shift work, regularly travel across time zones and have irregular habits. It is reasonable to infer that text materials reflecting individuals’ daily routines and life states are appropriate resources for understanding and analyzing individuals’ daily rhythms of emotions.

The dynamic assessment method is most commonly used to assess the daily rhythms of emotion; ecological momentary assessment, experience sampling method, diary methods (Bolger et al., 2003), and day reconstruction method are some examples of dynamic assessment methods. In a previous study, using these methods, participants were asked to assess their emotional states at different time points; thus, the trajectory of an individual’s emotion with changes in time and situation was drawn (Zhang et al., 2016). Specifically, using an ecological momentary assessment, participants repeatedly recorded their mental state and behavior in a natural environment with portable devices; thus, the researchers obtained data about individuals’ states in real-time (Shiffman et al., 2008). As there is no recall bias in the ecological momentary assessment, it has high ecological validity. In a study using the day reconstruction method, participants were asked to recall the previous day’s activities through questionnaires and assess the emotional states and experiences associated with the activities. As the activities had a timestamp, individuals’ daily rhythms of emotion, state, and other aspects were obtained (Kahneman et al., 2004).

However, in the methods mentioned above, individuals reacted to the assessment itself, which may lead to reactive bias. With the development of big data technology and social networks, researchers have begun to study individuals’ rhythms of emotion by analyzing big data from social networks (Wang et al., 2016; Eichstaedt and Weidman, 2020).

The data formed on social networking sites are naturally occurring and abundant. They provide a new, non-intrusive method to understand users’ mental states (Birnbaum, 2004) in which defensive thinking, social desirability effects, and memory bias can be avoided. In a study that included data from undergraduate students in the United States and Germany, Back et al. (2010b) found that data from social networks reflected participants’ mental states and personality traits. Further, data from social networks are tagged with time-stamps, making it possible to monitor and retrospectively examine the progress of mental state over time. Back et al. (2010a) explored the influence of terrorist attacks on people’s emotions by analyzing text content in their mobile phone messages before and after 9/11. Since text messages are time-stamped, emotional changes over time can be analyzed retrospectively.

A common way of excavating and analyzing data from social networks is to extract the features of text based on word frequency statistics and emotional tendency analysis (Cao et al., 2018) which were widely used in psychological studies (Liu et al., 2018; Sterling et al., 2020). This method is based on mental dictionaries. The most widely used mental dictionary is the Linguistic Inquiry and Word Count dictionary, which is appropriate for English text (Tausczik and Pennebaker, 2010). The dictionaries appropriate for analyzing simplified Chinese text are those in the Chinese mental analysis system (hereinafter called the “Wen Xin”) (Zhu, 2016) and HowNet (You et al., 2013). HowNet includes only positive and negative emotions. Alternatively, the dictionary in the “Wen Xin” system includes a fairly large number of categories of emotions; however, it was constructed based on the data from micro-blog specifically, and therefore, may not be appropriate for data analysis for other social networks.

Some researchers doubt the accuracy of text analysis based on word frequency and emotional tendency analysis, as text analysis does not consider irony, sarcasm, or unusual sentence structures (Pennebaker et al., 2003). However, Kahn et al. (2007) insisted on the validity of text analysis. According to their experiments, there was a significant correlation between the number of emotion words in a text based on the Linguistic Inquiry and Word Count dictionary and ratings for emotion by judges who analyzed the same text. This result indicates that text analysis, based on word frequency, is a valid reflection of an individual’s emotional state. Further, although irony and sarcasm may impact a specific sentence, they may offset each other when analyzing massive texts. Another study in China found that the frequency of a specific emotion word in a text was highly correlated with the human evaluation of emotion expression in that text (Zhao et al., 2016). The researchers also found that the efficiency of the Simple Chinese Linguistic Inquiry and Word Count dictionary in classifying emotions differed based on the type of text, with higher efficiency in news comments and RenRen Blog text and lower efficiency in micro-blog text. In general, text analysis based on the frequency of emotion words is valid for studying individuals’ emotions. It is worth noting that emotion dictionaries should be appropriated for different types of text.

For undergraduates in China, the most frequently used social networking site is Tencent QQ, which is similar to Facebook. Due to its multifunctionality, diversity, and personalized customization, Tencent QQ has become one of the most popular social networks among undergraduates (Zhang and Li, 2009). Tencent tweets (TTs), which are similar to the Facebook timeline, are the main way Chinese undergraduates post about their life situations and express their emotions and feelings. Therefore, the present study used the text analysis method to analyze text from TTs and explore the daily rhythms of undergraduate students’ emotions.

Undergraduate students’ emotions are influenced by demographic characteristics. Those who were once left behind in childhood had a higher rate of being diagnosed with depression (Han et al., 2017). Moreover, students from divorced families showed significantly higher levels of depression and anxiety (Huang and Li, 2011). Furthermore, female undergraduates showed more anxiety than male undergraduates, and those who reported high economic pressure had more emotional disorders (Stallman, 2010). Therefore, the other purpose of the present study is to examine whether there are differences in the daily rhythms of emotion among different demographic groups.

The present study explores the daily rhythm of undergraduate students’ emotions by analyzing TTs. This not only enriches the emotional analysis research of social media texts other than Twitter, Facebook, or micro-blog, but also provides new evidence for undergraduate students’ emotion expression on social networks within Chinese collectivist culture. This study has the potential to help students improve their emotional regulation abilities.

## Materials and Methods

### Participants

During the period from September to November 2018, we recruited participants by posting advertisements in TT online and displaying the advertisements on a PowerPoint Presentation during break time in mental health classes at the college. The participants needed to add the researchers’ Tencent QQ and allow the researchers to visit their TTs, which is an application of Tencent QQ. They also completed a questionnaire about their demographic information online. After removing Tencent QQ users who belong to social groups or engage in posting advertisements and those without data, a total of 894 Tencent QQ users were recruited for our research. Specifically, there were 554 female students (61.97%), 340 male students (38.03%), 425 freshmen (47.54%), 320 sophomores (35.79%), 125 juniors (13.98%), and 24 seniors (2.68%). The numbers of students from rural areas, towns, and cities were 411 (45.97%), 264 (29.53%), and 219 (24.50%), respectively; 306 students (34.23%) were from single-child families, and 588 (65.77%) were from multiple-child families. There were 807 students (90.27%) whose parents had a healthy and harmonious relationship and 87 students (9.73%) who came from broken families. Further, 375 students were left behind in childhood (41.95%). There were 121, 168, 242, 234, 89, and 40 students whose family economic conditions allowed them to afford tuition and living expenses very easily (13.53%), fairly easily (18.79%), easily (27.07%), difficult (26.17%), fairly difficult (9.96%), and very difficult (4.47%), respectively.

### Crawling and Preprocessing Tencent Tweet Text

#### Tencent Tweet Text Crawling and Segmentation

Texts from participants’ TTs from September 1, 2018 to August 31, 2019, were analyzed using a self-written Python language program. A total of 45,996 TTs from 894 participants were crawled and stored with timestamps. All the text was segmented into words by Jieba, a Chinese word segmentation tool (Sun, 2020); 37,91,618 words were obtained.

#### The Construction of an Emotion Dictionary Suitable for Tencent Tweet Text

We constructed a dictionary to process TT text information based on word frequency using three lexicons: the Wen Xin system, the HowNet system, and the TT text itself.

We constructed the dictionary as follows. First, two experts with years of work experience in mental health discussed and formed the following rules of classification: (1) each word is first classified into two categories, that is, positive emotion and negative emotion, followed by the secondary emotion category. The secondary emotion category of positive emotion included joy, pride, satisfaction, love, and gratitude, and that of negative emotion included depression, anxiety, anger, and fear. (2) The word can be assigned to a certain category if it is directly or indirectly related to that category. Second, the rules were presented to four senior year students majoring in psychology, who were asked to classify all the words independently, according to the rules. Third, if a word was assigned to the same category by three or more students, it was determined to be assigned to that category; if a word was assigned to a category by one or two students, a discussion was held to determine the category of that word. However, there were still 19 words for which they failed to reach an agreement after discussion. Then, 226 undergraduate students were recruited online to investigate which category those unclassified words belonged to. According to the results of the investigation, each word belonged to the corresponding emotional category to which most students classified it. The final constructed dictionary and its relationship with the lexicons taken as resources are listed in Table 1.

**TABLE 1:** The constructed dictionary suitable for TT text.

Category of emotion	Words account	Number of words overlapping with		
		“Wen Xin”	HowNet	high-frequency words from TT
Positive emotion	563	170	419	50
happiness	151	33	108	15
pride	43	10	27	3
satisfaction	148	32	121	11
love	125	46	84	16
gratitude	33	10	22	4
Negative emotion	1078	305	854	32
depression	132	37	106	3
anxiety	232	68	187	8
anger	344	90	270	11
fear	110	23	95	2

To further examine the accuracy of the constructed dictionary, three postgraduates majoring in psychology reassigned all the words according to rules, and their results were compared with the results of the four undergraduate students. For the emotion categories, the consistency ratio of the two results was 79.71–98.04%, while the total non-consistency ratio was 0.79–1.59%. There was high consistency between the results; therefore, the constructed dictionary was deemed reliable for TT text.

#### Counting and Storing Emotion Words in Tencent Tweet

We counted the total number of words in every TT text and the number of emotion words based on the constructed dictionary using a self-written Python language program. These were stored for further analysis.

### Data Analyzing Steps

First, we counted the total number of words in the TT text and the amount of TT text posted per hour (*h*) by each user (*u*). We then calculated the average number of words in a TT text, which is regarded as the index for the students’ activity degree in TT.

Second, regarding every single TT text posted by the user (*u*) as the smallest unit, we obtained the frequency of emotion words for a certain category by dividing the number of emotion words for a certain category *(AWORDS)* by the total number of words of the same TT *(WORDS)*:


$$A_{u}=\frac{A\,W\,O\,R\,D\,S_{u}\,\left(n\right)}{W\,O\,R\,D\,S_{u}\,\left(n\right)}$$

Then, we calculated the average frequency of emotion words for a certain category within a specific hour.


$$A_{u}\,\left(h\right)=\bar{A_{u}}=\frac{1}{N}\,\sum_{n\in N}\frac{A\,W\,O\,R\,D\,S_{u}\,\left(n\right)}{W\,O\,R\,D\,S_{u}\,\left(n\right)}$$

Third, we analyzed the daily rhythms of different emotions in TT and individual differences in daily rhythms using HLM6.2 to establish a multilevel linear regression model. The details are as follows.

Three models were successively constructed. As the initial word frequency was so small, we used the adjusted word frequency, which equals 1,000 times the initial word frequency, as the dependent variable in the three models. First, we established a null model; the ICC of the null model was calculated to determine whether a two-level model was necessary. Second, the random-effects model (Model 1), where the time t (0, 1, 2…23) was taken as the first-level independent variable, was established to explore the trend of frequency of emotion words for a specific category over time. Third, based on Model 1, the demographic variables were added as second-level independent variables to investigate how the frequency of emotion words for a specific category and their daily rhythms vary among different groups. Finally, a simplified model (Model 2) was obtained by retaining only the significant demographic variables. If Model 2 could not explain more random effects than Model 1, Model 1 would be the final model.

## Results

### The Degree of Activity in Tencent Tweets

**FIGURE 1:**
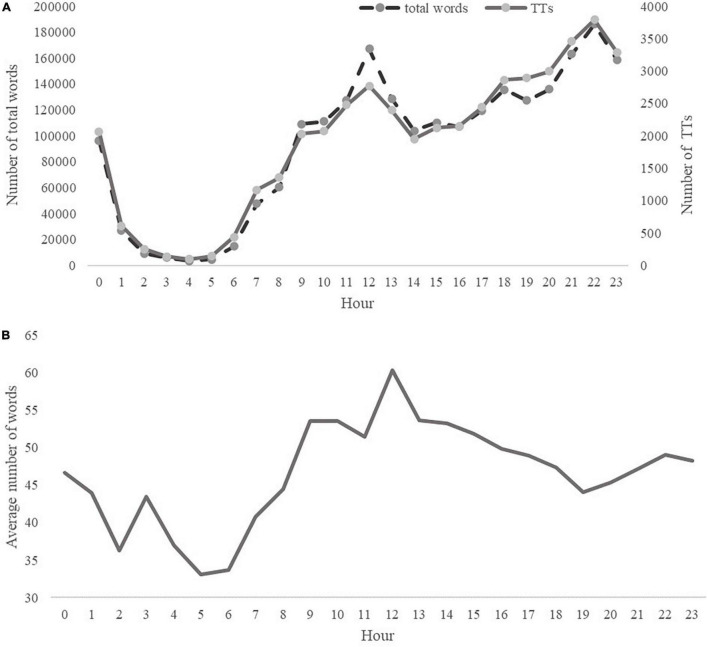
**(A)** Daily rhythms of the number of total words and TTs. **(B)** Daily rhythms of the average number of words per TT.

According to the total number of words and the number of TTs (Figure 1A), we can see a trough and two peaks on the curve. The trough was located between 1 a.m. and 6 a.m. when most students were sleeping. The two peaks were located at 12 a.m. and 10 p.m. The total number of words reached its highest point at 10 p.m.

When turning to the average number of words per TT in an hour, we can see only one peak and a trough (Figure 1B). The average number of words per TT reached the highest point at 12 a.m., indicating that TTs posted at this moment contain more information. What calls for special attention is that the number of TTs posted at 10 p.m. was large, but the average number of words per TT was not. The average number of words per TT in an hour reached its lowest point at 5 a.m. to 6 a.m. Although the number of TTs posted from 1 a.m. to 4 a.m. was also at its lowest point, the average number of words per TT was not very low.

### Daily Rhythms of Emotion

**FIGURE 2:**
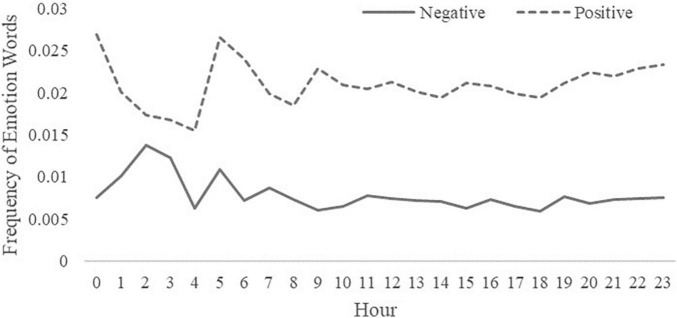
The daily rhythm changes of positive emotions and negative emotions.

**FIGURE 3:**
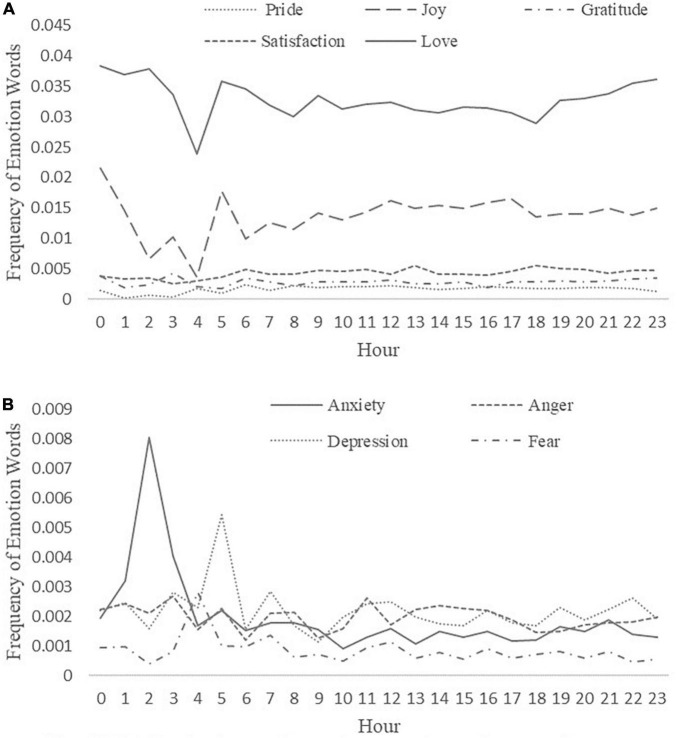
**(A)** Daily rhythms of positive emotion subcategories in TTs. **(B)** Daily rhythms of negative emotion subcategories in TTs.

The daily rhythms of emotion according to the frequency of positive and negative emotion words are shown in Figure 2. The daily rhythms of the subcategory emotions are shown in Figure 3. The frequency of positive emotion words (0.0209) was about three times that of negative emotion words (0.0078), which means that students expressed much more positive emotion in TTs. It is worth noting that from 1 a.m. to 4 a.m., there was a notable increase in the frequency of negative emotion words and a decrease in the frequency of positive emotion words.

Figure 3 shows that the word frequency of love (0.0328) was higher than other subcategories of positive emotion (pleasure: 0.0137, gratitude: 0.0029, satisfaction: 0.0043, and pride: 0.0017). Additionally, the word frequencies of satisfaction, gratitude, and pride changed little over time. There was a trough of the word frequency of pleasure from 3 a.m. to 5 a.m. Among the subcategories of negative emotion, the word frequencies of anger, depression, and anxiety were similar (0.0020, 0.0022, and 0.0020, respectively), while the word frequency of fear was much lower (0.0009). The peak of the curve for all subcategories of negative emotion accrued from 1 a.m. to 6 a.m. The results revealed that the word frequencies of different emotions in TTs changed differently over time.

### Intragroup Difference in Daily Rhythms of Emotions

#### The Variance for the Word Frequency of Emotion in Tencent Tweets

The ICC in the null model for different types of emotions was greater than 0.059 (Table 2), which means that the individuals’ variation in second-level accounted for a large enough proportion of the total variation. Therefore, a multilevel linear model was necessary (Fang et al., 2013). Compared with negative emotion and its subcategories, individual variation in second-level accounted for a larger proportion of the total variation for positive emotion and its subcategories.

**TABLE 2:** ICC in the null model for emotions.

Category of emotions	ICC
Positive emotion	0.289
pleasure	0.322
gratitude	0.143
satisfaction	0.189
pride	0.194
love	0.360
Negative emotion	0.176
anxiety	0.073
fear	0.073
anger	0.106
depression	0.062

#### The Trends of Word Frequency of Emotions Over Time in Tencent Tweets

As shown in Model 1 in Tables 3, 4, time had a significant predictive effect on the word frequency of each emotion in undergraduate students’ TTs; that is, the word frequency of each emotion in TTs had a significant increasing trend during the day. The slopes of time for positive emotion and its subcategories ranged from 0.260 to 4.051 (*ps.* < 0.001), among which the slopes for love and pleasure were much steeper (4.051 and 1.571, respectively). Additionally, the slopes of time for negative emotion and its subcategories ranged from 0.060 to 0.875 (*ps.* < 0.001), among which the slope for depression was much steeper (0.308).

**TABLE 3:** Multilevel linear model of positive emotion and its subcategories.

																	
Emotions	Positive emotion			Pleasure			Gratitude			Satisfaction			Pride		Love		
Predictive variable	Null	1	2	Null	1	2	Null	1	2	Null	1	2	Null	1	2	Null	1
Level-1																	
Intercept γ_00_	76.034*** (20.232)	75.284*** (20.343)	75.215*** (20.496)	49.434*** (11.313)	48.883*** (11.452)	48.809*** (11.578)	11.427*** (18.880)	11.317*** (18.825)	11.388*** (19.133)	16.746*** (15.301)	16.522*** (15.526)	16.530*** (15.529)	6.480*** (11.744)	6.393*** (11.818)	112.632*** (19.411)	111.325*** (19.564)	111.287*** (19.742)
Slope-time γ_10_		2.748*** (9.193)	2.747*** (9.077)		1.571*** (4.545)	1.563*** (4.532)		0.428*** (7.257)	0.549*** (8.430)		0.670*** (6.594)	0.667*** (6.597)		0.260*** (5.789)		4.051*** (9.210)	4.096*** (9.255)
Level-2																	
predictive variables of intercept																	
Gender γ_01_									4.006** (3.569)								
Grade γ_02_			−10.308* (−2.059)						−1.567* (−2.409)								
Origin γ_04_			16.744** (3.157)														13.281* (2.362)
Economic status γ_06_						−8.254* (−2.081)											
predictive variables of slope (interaction)																	
Grade γ_12_			−1.049** (−2.954)						−0.273** (−3.533)								−1.102** (−2.917)
Origin γ_14_			1.019** (2.748)														
Family structure γ_15_												0.518* (2.340)					
Economic status γ_16_						−0.594* (−2.567)											
**Random effects**																	
τ_00_	9459.071***	9461.616***	9239.615***	13196.850***	13078.276***	12972.901***	192.460***	193.645***	188.613***	698.796***	696.595***	696.563***	179.432***	178.444***	23987.867***	23902.104***	23578.552***
τ_11_		31.346***	30.245***		53.756***	53.213***		0.576			4.218***	4.199***		0.685		84.824***	83.413***
σ^2^	23273.814	20353.182	20354.820	27730.023	22955.993	22958.177	1149.844	1107.640	1135.764	3006.871	2660.131	2659.810	744.649	691.387	42593.296	34614.048	34624.662
R^2^_*level–1*_		12.45%			17.22%			3.67%			11.53%			7.15%		18.73%	

*p < 0.05; **p < 0.01; *** p < 0.001.

**TABLE 4:** Multilevel linear model of negative emotion and its subcategories.

Emotions	Negative emotion			Anxiety			Fear			Anger			Depression		
Predictive variable	Null	1	2	Null	1	2	Null	1	2	Null	1	2	Null	1	2
Level-1															
Intercept γ_00_	25.818*** (17.320)	25.633*** (17.429)	25.592*** (17.611)	5.864*** (13.600)	5.843*** (13.591)	5.851*** (13.706)	2.867*** (10.304)	2.857*** (10.285)	2.853*** (10.389)	6.968*** (12.544)	6.907*** (12.556)	6.921*** (12.691)	8.124*** (14.605)	8.035*** (14.711)	8.093*** (14.839)
Slope-time γ_10_		0.857*** (6.051)	0.854*** (6.135)		0.123* (2.401)	0.163** (2.944)		0.060* (2.467)	0.073** (2.877)		0.241*** (4.022)	0.367*** (5.209)		0.308*** (4.549)	0.441*** (5.631)
Level-2															
predictive variables of intercept															
Grade γ_02_			−3.630^†^ (−1.918)												−2.133** (−3.232)
Single-child γ_03_												4.082** (3.209)			
Origin γ_04_			4.640* (2.550)						0.676* (2.149)						1.407* (2.048)
Economic status γ_06_						−0.733* (−2.228)									
predictive variables of slope (interaction)															
Gender γ_11_												0.289* (2.071)			
Grade γ_12_			−0.405* (−2.481)												−0.228** (−2.615)
Single-child γ_13_			0.603* (2.165)			0.290** (2.664)			0.130* (2.497)						
**Random effects**															
τ_00_	1264.451***	1265.218***	1244.451***	70.801***	71.534***	70.205***	29.528***	29.989***	29.338***	142.271***	142.463***	139.217***	107.616***	108.839***	104.427***
τ_11_		5.083***	4.905***		0.262			0.031			0.554			0.780	
σ^2^	5907.974	5503.296	5503.080	904.027	890.143	902.119	376.649	374.491	376.306	1197.128	1157.857	1190.915	1639.660	1588.305	1630.965
R^2^_*level–1*_		6.85%			1.54%			0.57%			3.28%			3.13%	

^†^p < 0.1; *p < 0.05; **p < 0.01; ***p < 0.001.

According to the random effects in Model 1, the proportions of variation of positive emotion and its subcategories, which could be explained by the time, were between 3.67 and 18.73%. In comparison, the proportions of variation of negative emotion and its subcategories were between 0.57 and 6.85%. Overall, positive emotion and its subcategories fluctuated more over time, while negative emotion and its subcategories were more stable.

#### Intragroup Difference in Word Frequency and Daily Rhythms of Emotions

As shown in the final model in Tables 3, 4, the word frequency and daily rhythms of emotions, derived from the TTs, varied differently depending on the demographic variables, except for the variable of left-behind during childhood.

(1) **Gender.** There was not much difference in the frequency and daily rhythms of emotion words between male and female students in their TTs. A significant difference existed only in word frequency of gratitude (γ_01_ = 4.006, *p* < 0.001) and the daily rhythms of anger (γ_11_ = 0.289, *p* < 0.05). The word frequency of gratitude in female students’ TTs was higher than that of male students. The word frequency of anger varied much more in female students’ TTs than in male students’ TTs during a day.
(2) **Grades.** As the grade increased, the word frequency of positive emotion (γ_02_ = −10.308, *p* < 0.05), gratitude (γ_02_ = −1.567, *p* < 0.05), and depression (γ_02_ = −2.133, *p* < 0.01) in TTs decreased significantly. The higher the grade, the slower the word frequency of positive emotion (γ_12_ = −1.049, *p* < 0.01), gratitude (γ_12_ = −0.273, *p* < 0.001), love (γ_12_ = −1.102, *p* < 0.01), negative emotion (γ_12_ = −0.405, *p* < 0.05), and depression (γ_12_ = −0.228, *p* < 0.01) increased over time.
(3) **Single-child and multiple-child.** Students in single-child families had a higher word frequency of angry (γ_03_ = 4.082, *p* < 0.01) compared to those with siblings. The word frequency of negative emotion (γ_13_ = 0.603, *p* < 0.05), anxiety (γ_13_ = 0.209, *p* < 0.01), and fear (γ_13_ = 0.130, *p* < 0.05) in only children’s TTs had a higher rate of change with time. There were remarkable differences in the word frequency of negative emotion and its subcategories in TTs between students in single-child families and students with siblings, while there were no differences in the word frequency of positive emotion and its subcategories.
(4) **Place of origin**. With the changes in students’ birthplace from rural areas, towns, and cities, the word frequency of positive emotion (γ_04_ = 16.744, *p* < 0.01), love (γ_04_ = 13.281, *p* < 0.05), negative emotion (γ_04_ = 4.640, *p* < 0.05), fear (γ_04_ = 0.676, *p* < 0.05), and depression (γ_04_ = 1.407, *p* < 0.05) increased, which indicated that students from cities were more demonstrative and showed positive and negative emotions through TTs. Moreover, the frequency of positive emotion words in urban students’ TTs increased rapidly over time (γ_14_ = 1.019, *p* < 0.01), indicating that the fluctuation of positive emotion in urban students was greater during the day.
(5) **Family structure and economic status.** Family structure was related to the daily rhythms of satisfaction in students’ TTs (γ_15_ = 0.518, *p* < 0.05). Students whose parents were alive and had a healthy marital relationship had more intense variations in satisfaction during the day. The word frequency of pleasure (γ_06_ = −8.254, *p* < 0.05) and anxiety (γ_06_ = −0.733, *p* < 0.05) in TTs were also different for students with different family economic statuses. Specifically, the more difficult it was for students to pay for various expenses and the worse their family’s economic status, the lower the word frequency of pleasure and anxiety in their TTs. For students with different family economic statuses, there were also differences in the daily rhythms of pleasure (γ_16_ = −0.594, *p* < 0.05).

## Discussion

Many studies have been conducted on undergraduate students’ emotions; however, most investigated the states of their emotions rather than emotion fluctuation (Chen et al., 2016; Chester et al., 2016; Gloria and Steinhardt, 2016). In fact, emotional stability is important for individuals’ mental health. Researchers have examined dynamic changes in emotion based on social networks, using big data technology. Golder and Macy (2011) analyzed people’s diurnal and seasonal emotion rhythms using data from Twitter messages. Moreover, Wang et al. (2016) investigated emotion changes through data from Sina micro-blog, a popular social network in China. Instead of Twitter or micro-blog, Tencent QQ is the most widely used social network among Chinese undergraduates. They share their emotions and mental states with friends through TTs in Tencent QQ. Therefore, the present study analyzed TTs using big data technology to achieve three aims: (1) to determine whether TTs are suitable for the analysis of undergraduate students’ emotions; (2) to provide advice about emotion regulation strategies to undergraduates by exploring the status and rhythm of their emotions; and (3) to study intragroup differences in emotion status and rhythms.

Our results exhibited that the frequency of positive emotion words in TTs was much higher compared to negative emotion words. This finding is consistent with a general positive bias found in language; that is, the frequency of positive words is higher than that of negative words in both oral and written language (Dodds et al., 2015). However, there are more words to express negative emotions, both in existing dictionaries and the emotion dictionary constructed in this study. The frequency of posts about negative events in life was much lower than that of positive events, and thus the frequency of negative emotion words in the TTs was lower. Another possible reason for the lower frequency of negative emotion words is impression management; that is, undergraduates try to make a good impression on others by publishing more positive information on social networks (Xin et al., 2016).

In the positive emotion subcategories, the word frequency of love was much higher than that of other positive emotions, which is contrary to certain aspects of Chinese culture. Unlike the individualistic culture that encourages expressing feelings, the collectivist culture in China usually suppresses the free expression of emotions. That is to say, the Chinese are reluctant to express their negative feelings as well as one particular positive emotion—love. Previous studies have confirmed these findings. For example, compared to people from other cultures, Chinese people have been found to use emotion inhibition strategies more frequently and express fewer positive and negative emotions in emotional heuristic tasks (Soto et al., 2005; Wei et al., 2013). Moreover, they have been found to avoid expressing love (Caldwell-Harris et al., 2013). As far as we know, there are two explanations for the high word frequency of love in the TTs: the idealized virtual-identity hypothesis and the extended real-life hypothesis (Yao et al., 2014). The former holds that people tend to present an ideal self on social media; therefore, individuals are more likely to express positive emotions (Qiu et al., 2012). To create a good self-image, individuals are likely to express more love, which is a typical positive emotion, in TTs. The latter considers that social networks are an extension of real-life situations, and individuals will express their real selves on social media. Although Chinese individuals tend to be poor at expressing love in real situations, they do feel love and are more likely to express it on social media.

This study also found that undergraduates express depression, anxiety, and anger more than fear, which corresponds with their physical and mental characteristics and was consistent with the results of previous researches (Song and Fu, 2014; Cheng and Jia, 2019). Undergraduates are in the transition stage from adolescence to adulthood. In this important stage, they need to form a self-identity and face academic, interpersonal, emotional, economic, and other pressures. When individuals are unable to deal with these pressures, they are likely to experience depression and anxiety. The high word frequency of anger may be because many students lack new goals and are prone to boredom after starting university. Moreover, boredom is a hidden psychological state of suppression, which is an early sign of anger (Dahlen et al., 2004; Zhao et al., 2015). Therefore, undergraduates should make plans for college life and set reasonable goals to enrich their lives, avoid boredom, and reduce anger and aggressive behaviors.

The frequency of positive and negative emotion words had dynamic variations during a day, which synchronized with undergraduate students’ daily routines. The present study found that TT activity from 1 a.m. to 6 a.m. was low, and activity peaked at noon and about 10 p.m. This tendency is consistent with the results of Golder and Macy (2011), except that they found that Twitter activity peaked first at 9 a.m. rather than at noon. This inconsistency may be due to differences in the participants, as the participants of Golder and Macy’s (2011) research included not only undergraduates but many other groups as well. Most of them went to school or work and had limited time for social media after 9 a.m. Moreover, considering that the changes in individual emotions are closely related to their physiological rhythms and daily routines (Golder and Macy, 2011; Wang et al., 2016), the TTs reflecting undergraduate students’ life states can be important resources for analyzing their emotions.

There were intragroup differences in frequency and daily rhythms of emotion words. Grade was the main variable that affected the frequency of emotion words in TTs. As the grade increased, the frequency of emotion words in most subcategories in TTs decreased and the fluctuation in daily rhythms became smaller. We then performed an additional analysis about the user activity of Tencent QQ by students of all grades and found that both the average number of words and the average number of TTs per hour were the highest among freshmen, and the lowest among seniors and above. This result was consistent with previous research that senior students use social networks less frequently (Wang et al., 2015). However, the frequency of emotion words is an indicator excluding the influence of user activity of Tencent QQ. Therefore, the low frequency of emotion words in senior students’ TTs implied that senior students express less emotion on social networks. This result is similar to Lu et al. (2016), who found that students from higher grades express less emotion.

There was no significant difference in the frequency and daily rhythm of emotion words used in TTs based on having had a left-behind experience, which is inconsistent with existing research results (Li et al., 2009). The reason for this inconsistency may be that previous studies mostly used self-report scales to evaluate emotions, which is a retrospective evaluation induced by the outside world. In contrast, the data in this study came from TTs, which are spontaneous and real-time records that are not affected by recall bias. A study showed that the retrospective ratings of emotion are significantly higher compared to concurrent ratings for unpleasant conditions (Bruun and Ahm, 2015). Although there was no significant difference in emotion in daily life between undergraduates with or without a left-behind experience, it is possible that undergraduates with left-behind experience would have more retrospective emotional distress.

The present study explored the daily rhythms of emotions expressed by undergraduates in their Tencent tweets. To our knowledge, it is the first study to analyze word frequencies on a social network other than Twitter, Facebook, or micro-blog. Thus, this study has added new evidence that text on social media can be an important resource for analyzing undergraduates’ emotions. Adding to studies from individualistic cultures, the present study provides insights into emotion expression on social networks in a collectivist culture (China). The most important difference found in this respect is that Chinese individuals are more likely to express love on social media in comparison to real situations. In addition, the present study has also theoretical and practical implications.

The findings of the daily rhythms of positive and negative emotions showed that positive and negative emotions tended to have opposite trends from 12 a.m. to 2 a.m., 6 a.m. to 7 a.m., and 8 a.m. to 11 a.m. (i.e., as the frequency of one type of emotion word increased, the other decreased), and the same trend from 2 a.m. to 6 a.m., 7 a.m. to 8 a.m., and 2 p.m. to 9 p.m. (i.e., the frequencies of both positive and negative emotion words either increased or decreased). From 11 a.m. to 2 p.m. and 9 p.m. to 12 a.m., the trends were unrelated. These results are inconsistent with Russell’s (1980) circumplex model of affect, which considers that positive emotion and negative emotion are opposite poles of the valence dimension. According to the model, as one kind of emotion falls, the other rises. Our results support the positive and negative affect model proposed by Watson and Tellegen (1985) and the evaluation space model proposed by Cacioppo and Berntson (1994). According to Watson and Tellegen’s (1985) model, positive and negative emotions are two independent dimensions. Positive emotion is a combination of pleasure and high activation, while negative emotion is a combination of displeasure and high activation. According to the evaluation space model by Cacioppo and Berntson (1994), positive emotion and negative emotion are usually antagonistic to each other, but they may have uncoupled activation, co-activation, or co-inhibition. That is to say, a change in positive emotion may likely be the same as that in negative emotion, or it may occur in parallel or reverse with a change in negative emotion.

These notions provide directions for emotion regulation strategies. Previous researches focused on the strategies for decreasing negative emotion, such as reappraisal, suppression (Gross, 1998), and cognitive distraction (Strauss et al., 2016). However, our results suggested that the strategies of increasing positive emotion and controlling negative emotion are both essential when regulating emotions. Reappraisal, which was recently proved to be effective for decreasing negative emotion and increasing positive emotion, is a better strategy than distraction and suppression (Troy et al., 2019).

We found that the trough period for the average number of words in a single TT per hour was not from 1 a.m. to 6 a.m. but from 5 a.m. to 6 a.m. This means that the total number of words undergraduates published in a TT from 1 a.m. to 4 a.m. was low, but the average number of words per TT was high. The peak of the frequency of negative emotion words was from 1 a.m. to 4 a.m., and the trough of the frequency of positive emotion words was in the same period, which is consistent with previous research. Many studies have shown that late sleepers or those who have sleep disorders (i.e., individuals who are often awake from 1 a.m. to 4 a. m.) have high rates of depression, anxiety, and other emotional disorders (Alvaro et al., 2013; Zhang et al., 2017; Guo et al., 2018). Moreover, studies show that poor sleep quality can predict suicidal ideation (Supartini et al., 2016). Thus, it is reasonable to infer that undergraduate students who are active Tencent QQ users from 1 a.m. to 4 a.m. are likely to publish negative emotion words and are vulnerable to mental disorders. For students who are often active during the wee hours, more efforts should be made to adjust sleep rhythms and regulate emotions. It is also wise to seek help when necessary. The school administration department should also pay more attention to these students.

Our study is not without limitations. The first limitation was the representativeness of the sample as all the students were from a single university, and the number of students from the senior grade was less. The study should be replicated in the future using a more representative sample. Moreover, we did not explore the relationship between emotion expression on social networks and individuals’ mental health. Thus, it is still an open question whether emotion expression on social networks can predict mental health. Emotion expression may be a reflection of individuals’ current mental states, and emotion expression on social networks may be a strategy of emotion regulation that has positive impacts on individuals’ mental health. Further research should be conducted to determine the degree to which emotion expression on social networks is associated with mental health. Finally, text analysis based on word frequency in present study has been widely used and its validity was confirmed, but more text analysis methods have been developed, such as machine learning approach (Lee et al., 2019). These new methods should be alternative approaches to analyze the emotion in undergraduates’ texts on social media.

## Conclusion

Tencent Tweets provide important material for analyzing the state and variation of undergraduate students’ emotions. Undergraduates tweeted more positive emotions than negative emotions on TTs. The emotions in undergraduate students’ tweets changed considerably during the wee hours but were fairly stable during the day. Positive emotions increased more intensely than negative emotions during the day as each hour progressed. There were also intragroup differences in the use of emotion words in TTs. The late sleepers who are likely to post negative emotions should be paid close attention. As positive and negative emotions were not polar of one dimension, according to our results, strategies that act on both positive and negative emotions should be adopted when regulating emotions.

## Data Availability Statement

The raw data supporting the conclusions of this article will be made available by the authors, without undue reservation.

## Ethics Statement

Ethical review and approval was not required for the study on human participants in accordance with the local legislation and institutional requirements. The patients/participants provided their written informed consent to participate in this study.

## Author Contributions

Both authors designed the study, involved in data collection, and read and approved the final version of the manuscript. R-XL constructed the emotion dictionary for TT text. HL performed the statistical analysis. R-XL wrote the Introduction and Discussion sections of the first draft of the manuscript and HL wrote the remaining the sections.

## Conflict of Interest

The authors declare that the research was conducted in the absence of any commercial or financial relationships that could be construed as a potential conflict of interest.

## Publisher’s Note

All claims expressed in this article are solely those of the authors and do not necessarily represent those of their affiliated organizations, or those of the publisher, the editors and the reviewers. Any product that may be evaluated in this article, or claim that may be made by its manufacturer, is not guaranteed or endorsed by the publisher.

## Abbreviations

TT: Tencent Tweet.
